# Supplementary material for: Skin parasite landscape determines host infectiousness in visceral leishmaniasis
Source: Nat Commun. 2017 Jul 5;8:57. doi: 10.1038/s41467-017-00103-8 (PMC5498584; doi:10.1038/s41467-017-00103-8)
Supplement: Supplementary file 1 — Supplementary Information [file 41467_2017_103_MOESM1_ESM.pdf]

File name: Supplementary Information
Description: Supplementary Figures, Supplementary Tables, Supplementary Notes and Supplementary References

File name: Supplementary Software 1
Description: Source code and documentation for computational models

File name: Peer Review File
Description:

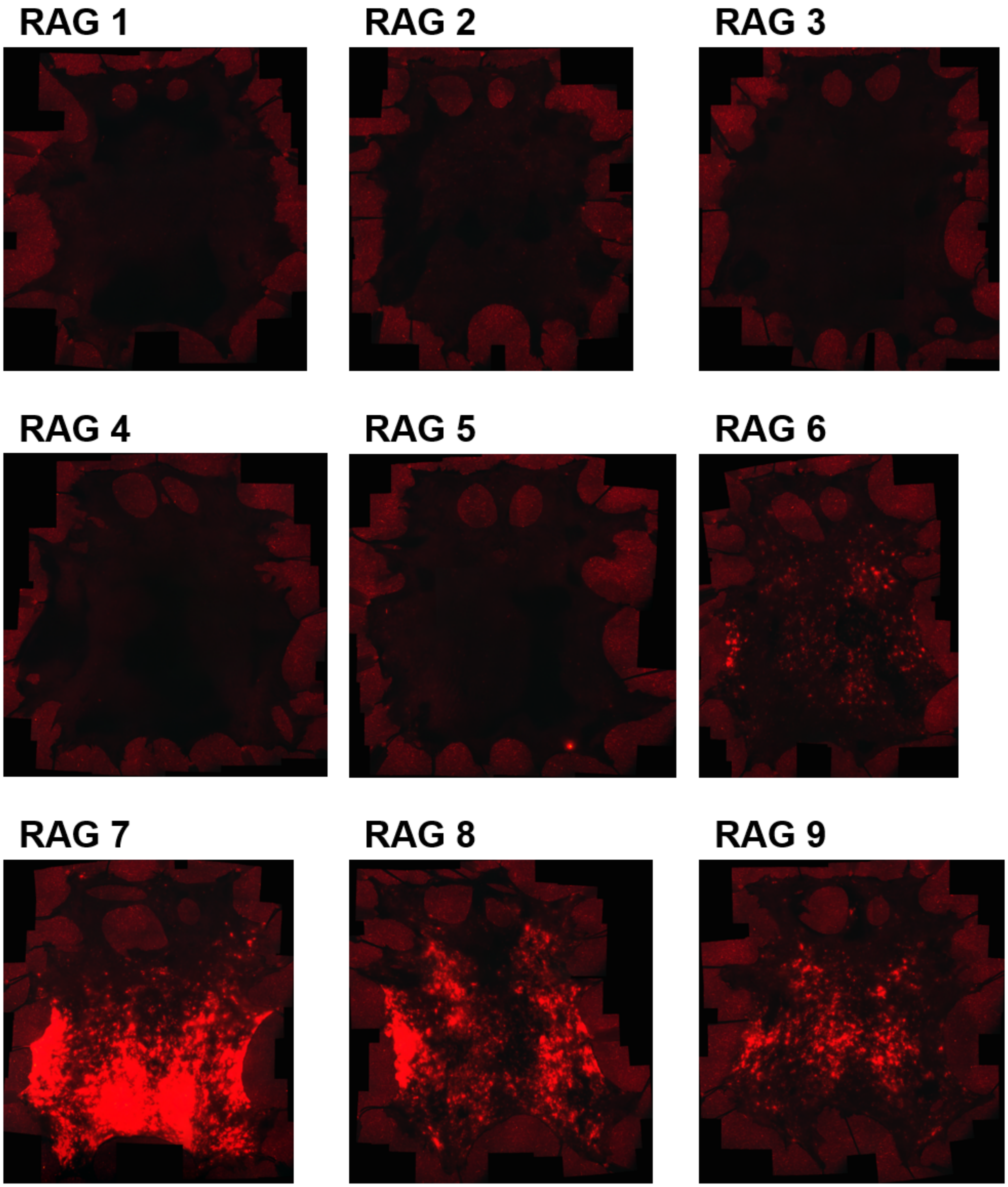

Supplementary Figure 1 | Stereomicroscopic composite images of skin from tdTom-*L. donovani* infected RAG$^{-/-}$ mice

Composite images of whole mouse skins are imaged from the hypodermal side with the same settings and exposure time. Therefore, the right-side flank is on the left-hand side of images and vice versa. Red fluorescence intensity is equal to parasite density in the skin.

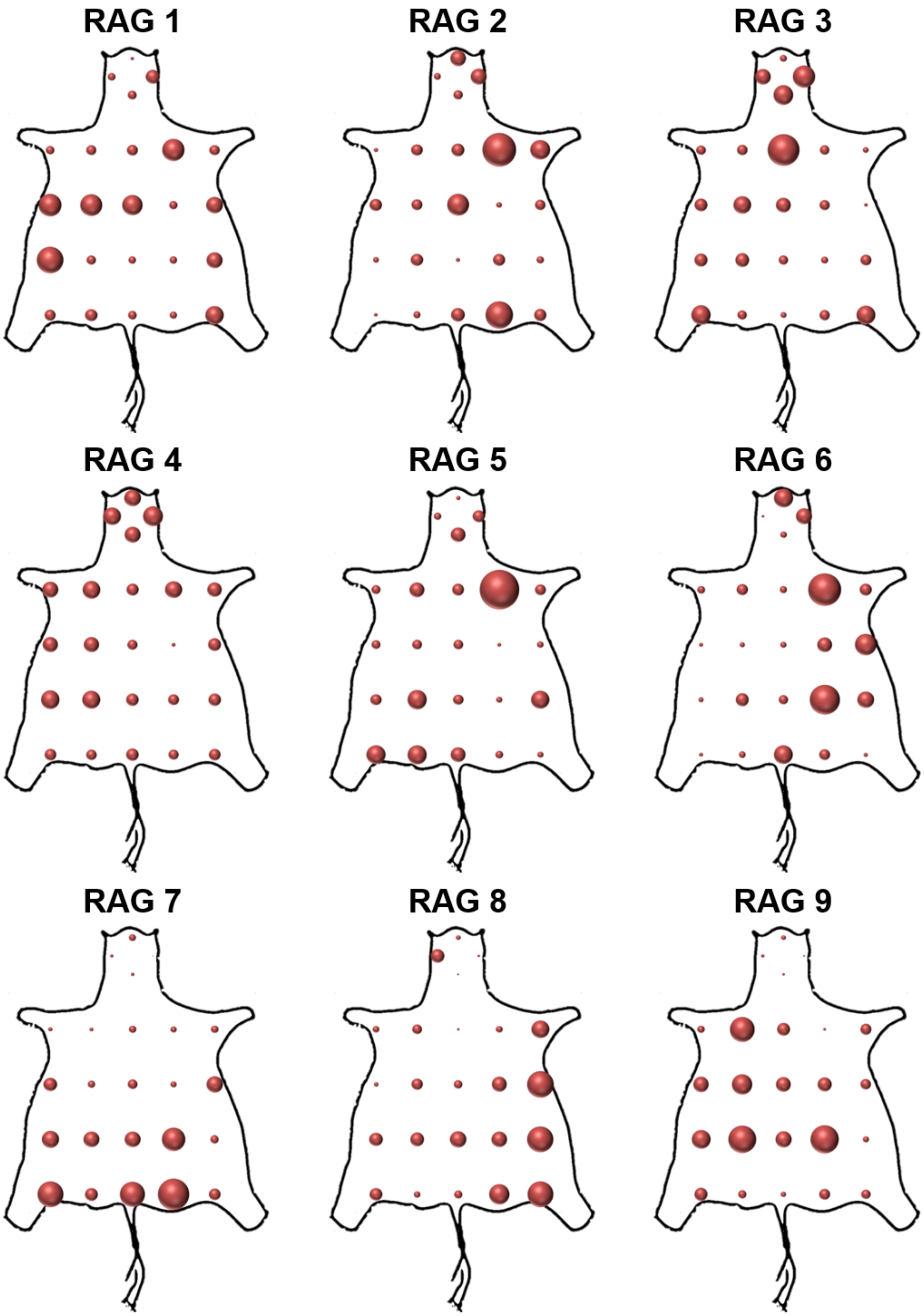

Supplementary Figure 2 | Bubble graphs of parasite load between punch biopsy sites in individual infected RAG$^{-/-}$ mice
The size of each bubble represents the proportion of total parasite load determined by qPCR in all biopsy sites (N=24), allowing comparison between mice. Bubble size indicates the distribution of parasites across the mouse skin as a means to identify preferential areas of parasite accumulation.

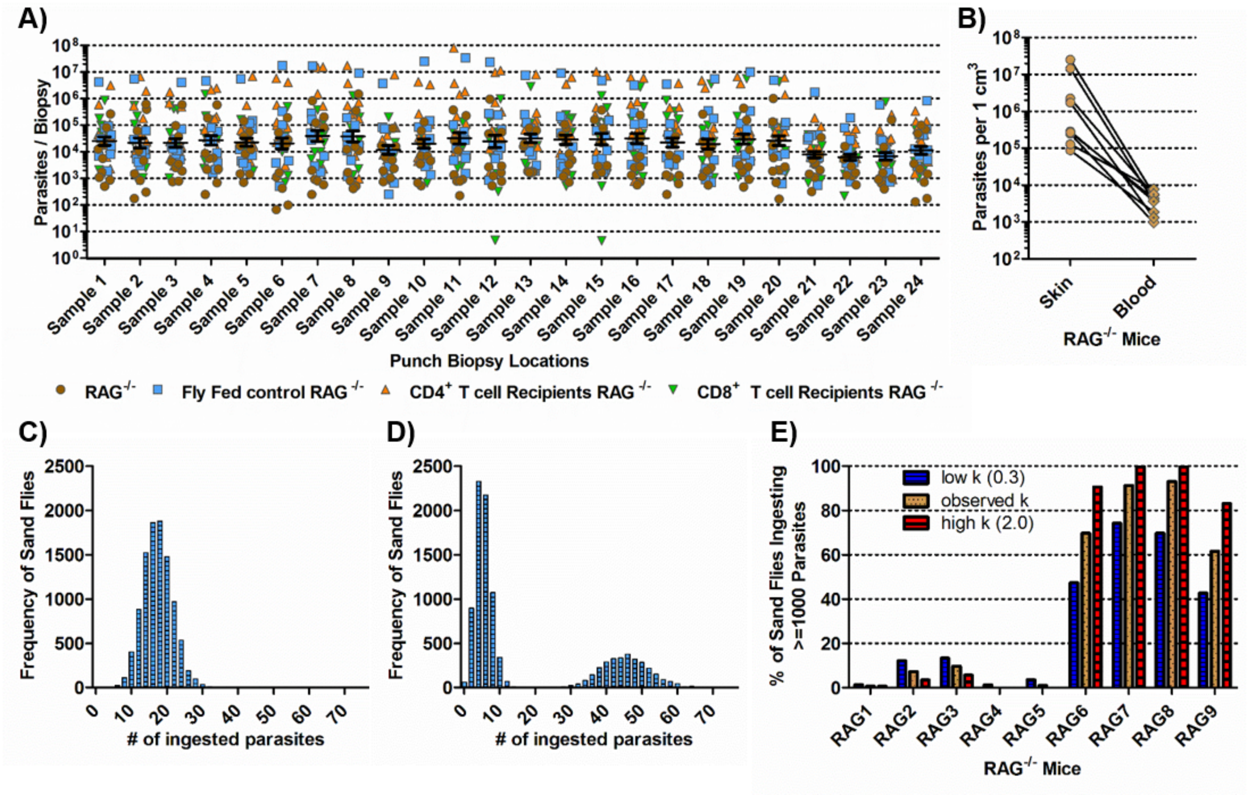

Supplementary Figure 3 | Analysing skin parasite load and distribution

This Figure is an extension of Fig.1. (A) Scatter plot for parasite load per biopsy location for all 24 biopsy locations. This figure is a repeat of Fig.1D, but includes the data from the RAG mice in Fig 1 as well as RAG mice receiving no transfer (Fly Fed control) or transfer of $CD4^+$ and $CD8^+$ T cells. Although the P-value for the overall Kruskal-Wallis test (P=0.025 [2-tailed]) was significant, the pair-wise comparison by 2-tailed Wilcoxon Signed Rank test of all sample sites did not show any significant differences between samples sites. This discrepancy may be due to the large sample variances and size (biological replicates per sample site N=36; total N=864). (B) Skin and blood parasite loads (biological replicates N=9 each) adjusted to 1 cm^3 volume for direct paired comparison of parasite load analysed by 2-tailed Wilcoxon Signed Ranks test (P=0.008). (C) Preliminary model prediction of parasite loads in 100,000 in silico sand flies from a homogeneous skin parasite distribution. (D) Corresponding model prediction of parasite loads in 100,000 *in silico* sand flies from a heterogeneous skin parasite distribution with the same mean. (E) In silico effect of adjusting *k*-values up (*k*=2) or down (*k*=0.3) on outward transmission potential in the preliminary model.

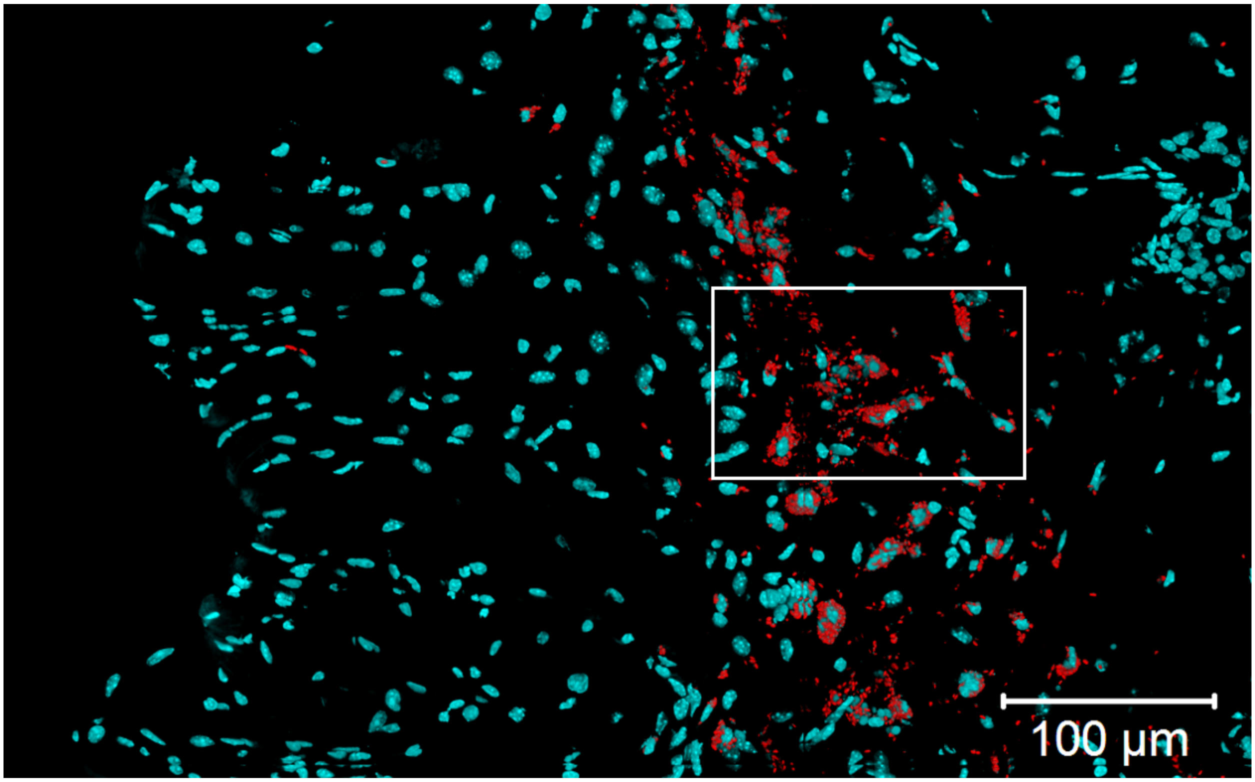

Supplementary Figure 4 | Confocal image of infected RAG mouse skin
The confocal image is a 3D rendition of Z-stack tile scan of a 10 µm thick skin section stained with DAPI (cyan) and containing tdTom-*L. donovani* (red). This image shows a skin area of higher parasite density and lower spread with $k \to 0$, akin to parasite patches. The white frame refers to the area of the images shown in Fig.1E.

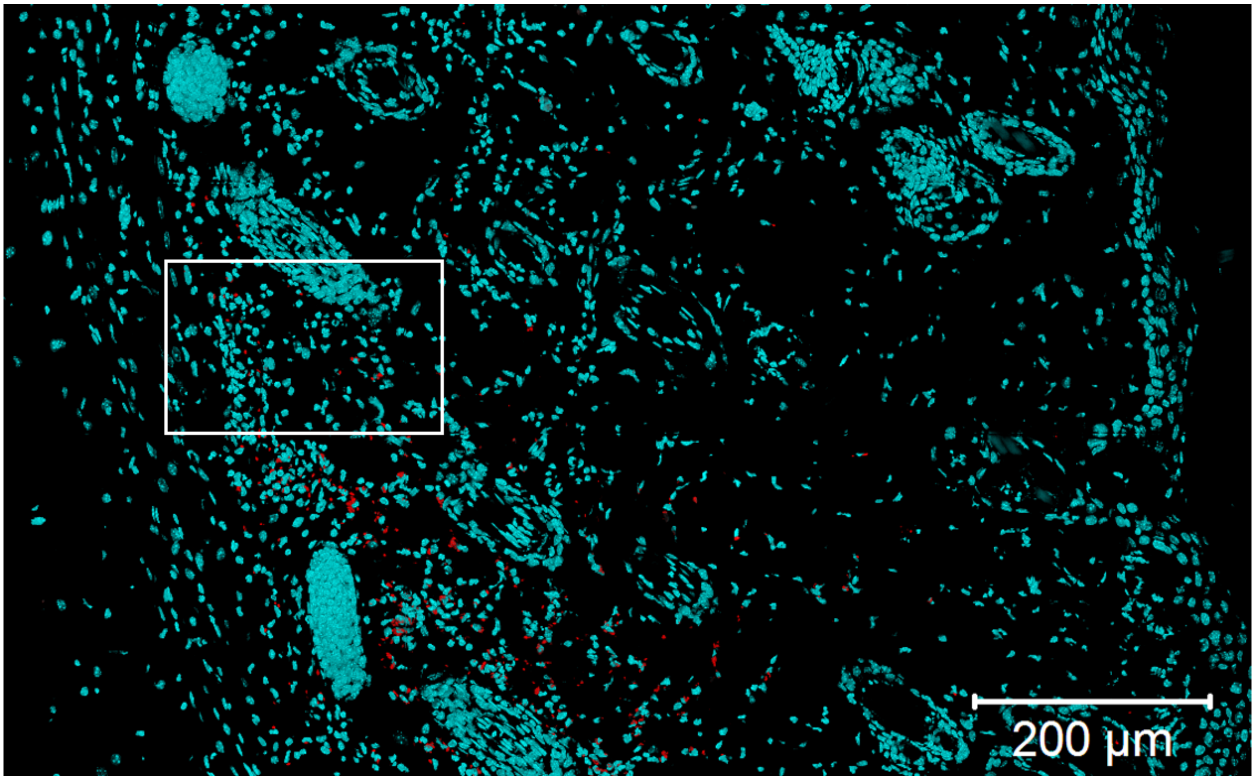

Supplementary Figure 5 | Confocal image of infected mouse skin
The confocal image is a 3D rendition of Z-stack tile scan of a 10 µm thick skin section stained with DAPI (cyan) and containing tdTom-*L. donovani* (red). This image shows a skin area of lower parasite density and greater spread with $k \to \infty$, moving toward a more homogeneous parasite distribution. The white frame refers to the area of the images shown in Fig.1F.

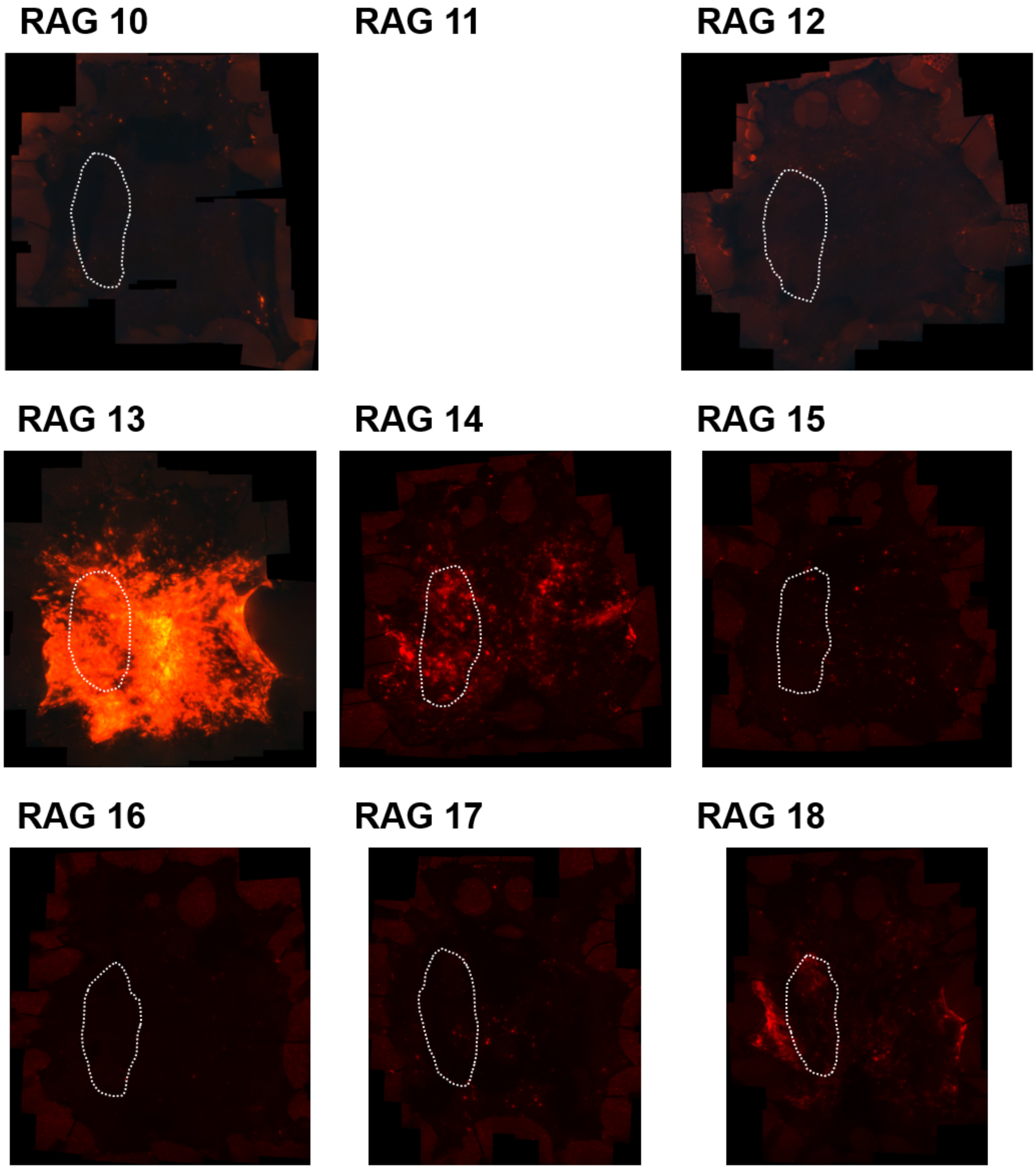

Supplementary Figure 6 | Stereomicroscopic composite images of infected "Fly Fed control" $RAG^{-/-}$ mice
Composite images of whole mouse skins are imaged from the hypodermal side with the same settings and exposure time. Therefore, the right-side flank is on the left-hand side of images and vice versa. Red fluorescence intensity is equal to parasite density in the skin. The broken white line outlines the sand fly exposed skin area. Images for Rag 11 are not available.

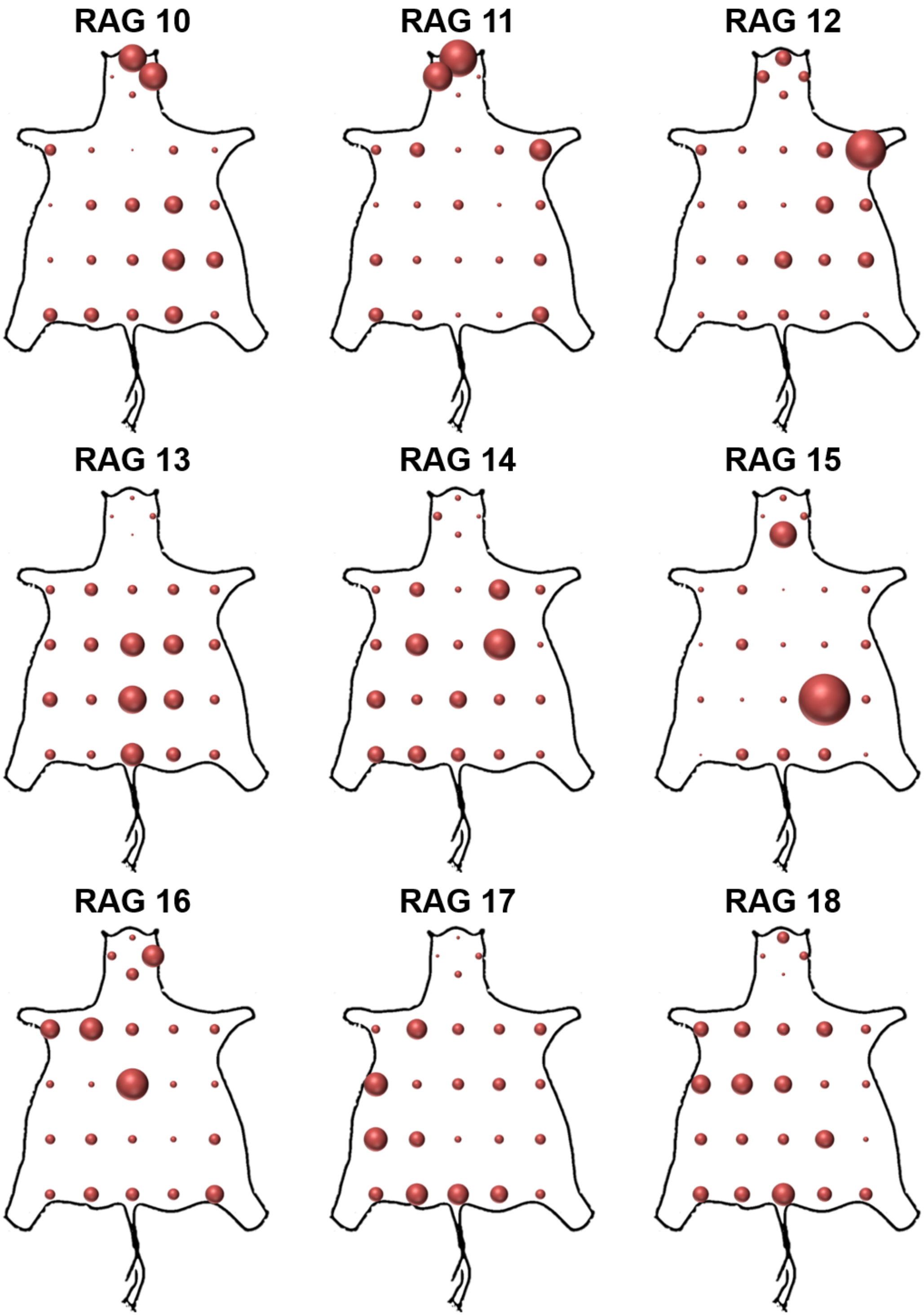

Supplementary Figure 7 | Bubble graphs of parasite load between punch biopsy sites in individual infected “Fly Fed control” RAG$^{-/-}$ mice
The size of each bubble represents the proportion of total parasite load determined by qPCR in all biopsy sites (N=24), allowing comparison between mice. Bubbles indicates distribution of parasites across the mouse skin as a means to identify preferential areas of parasite accumulation.

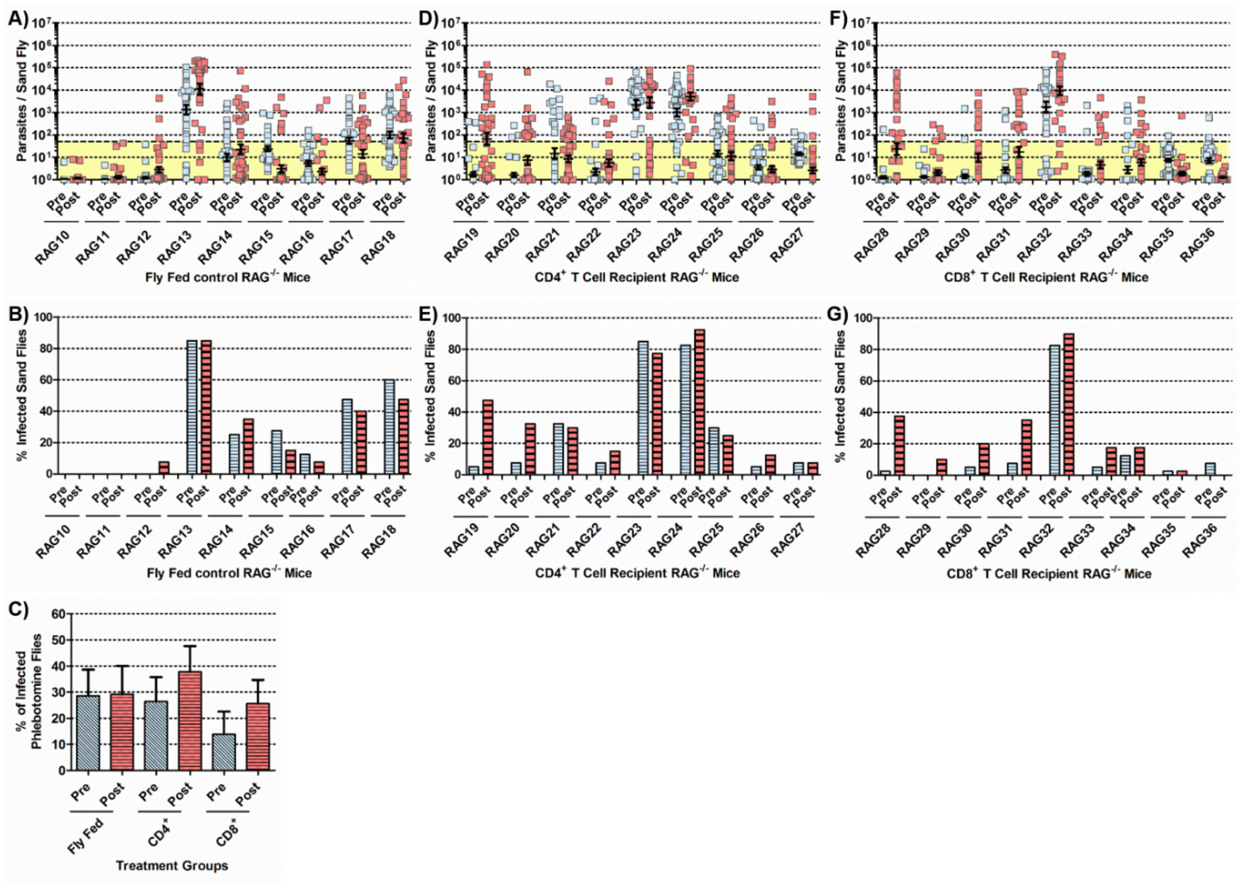

Supplementary Figure 8 | Sand fly parasites loads pre- and post-adoptive transfer in individual RAG mice

(A, D, F) Scatter plots of parasite load per sand fly for each individual mouse at day 7 PBM pre- (blue; first sand fly exposure; technical replicates N=40 per mouse, total N=360) and post-adoptive transfer (red; second sand fly exposure; technical replicates N=40 per mouse, total N=360) of either no cell (Fly Fed), naïve $CD4^+$ T cells ($CD4^+$) or naïve $CD8^+$ T cells ($CD8^+$). (B, E, G) Corresponding bar-charts of number of infected sand flies (harbouring >50 parasites) per exposed mouse. See Figure 6 for summary. (C) Comparison of sand fly infection success by feeding sand flies on mice of the Fly Fed, $CD4^+$ and $CD8^+$ groups pre- (first sand fly exposure; N=360 per group) and 9 days post-adoptive transfer (second sand fly exposure; N=360 per group). Standard Error bars are shown. 2-tailed Mann-Whitney U test: Fly Fed: $P=0.126$; $CD4^+$: $P=0.035$; $CD8^+$: $P<0.001$.

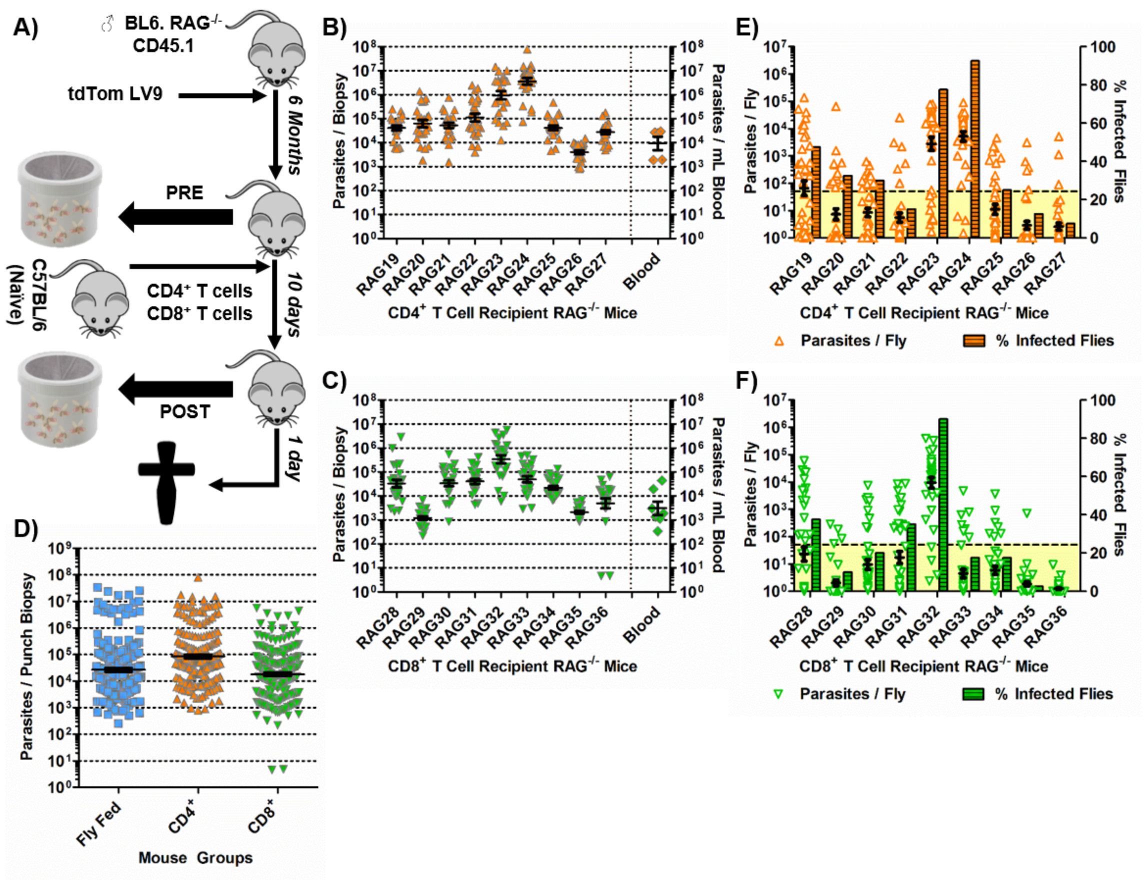

Supplementary Figure 9 | Analysing impact of skin parasite burden on outward transmission

This figure is an extension of Fig.2, showing the alternative CD4$^+$ and CD8$^+$ T cell recipient groups. (A) Schematic representation of the experimental sand fly exposure procedure. (B, C) Scatter plot of parasite loads in skin for each mouse (ø0.8 cm; vol. 12 mm^3; technical repeats N=24 biopsies per mouse; total N=216) and blood parasitemia (per ml; biological replicates N=9) of each mouse. Standard Error bars are shown. Skin parasite load variability per mouse was analysed by 2-tailed One-Sample t-test (P<0.001 each), data variance between all mice (biological replicates N=9) within the group by Brown-Forsythe test (P<0.001) and mean biopsy parasite load by one-way Kruskal-Wallis test (P<0.001). (D) Scatter plot comparing mean biopsy parasite load per group (mice: biological replicates N=9 per group, total N=27; biopsies: technical replicates N=24 per mouse, N=216 per group, total N=648) analysed by one-way Kruskal-Wallis test (see main text for P-values). (E, F) Experimental qPCR data of parasite loads per sand fly (second sand fly exposure; scatter plot; technical replicates N=40 per mouse, N=360 per group) paired with the ratio (%) of sand flies harbouring >50 parasites (threshold area in yellow) at day 7 post blood meal (PBM; bar chart). Standard Error bars are shown (Scatter plot only). Sand fly parasite load variability between sand flies fed on the same mouse were analysed by 2-tailed one-sample Wilcoxon Signed Rank test (P<0.001 each), sand fly data variance between all mice (biological replicates N=9 each group) within each group by Brown-Forsythe test (P<0.001) and mean sand fly parasite burden by one-way Kruskal-Wallis test (P<0.001 each group and between groups).

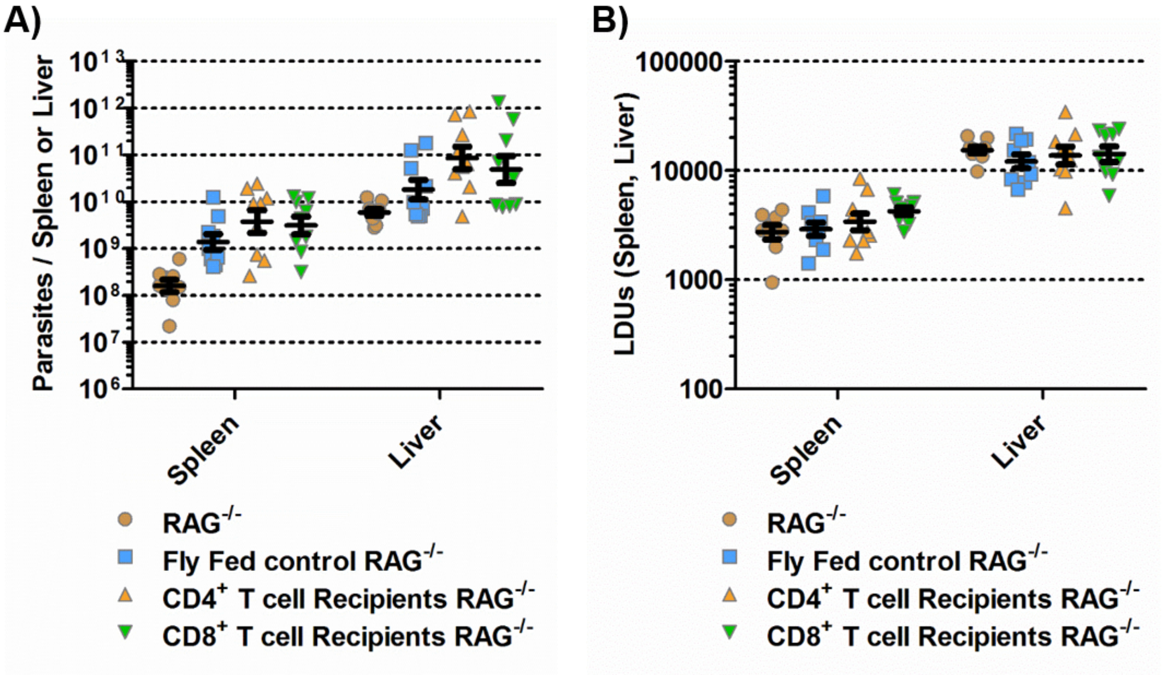

Supplementary Figure 10 | Tissue parasite loads as determined by qPCR and impression smear

Untreated mice were from a separate experiment and are not directly compared to the other groups. (A) Scatter plot of parasite load in spleen and liver based on qPCR data analysed by one-way Kruskal-Wallis test (see main text for P-values). (B) Scatter plot of spleen and liver parasite load according to Leishman Donovan Units (LDUs) analysed by one-way Kruskal-Wallis test (see main text for P-values). Biological replicates N=9 per group, total N=27.

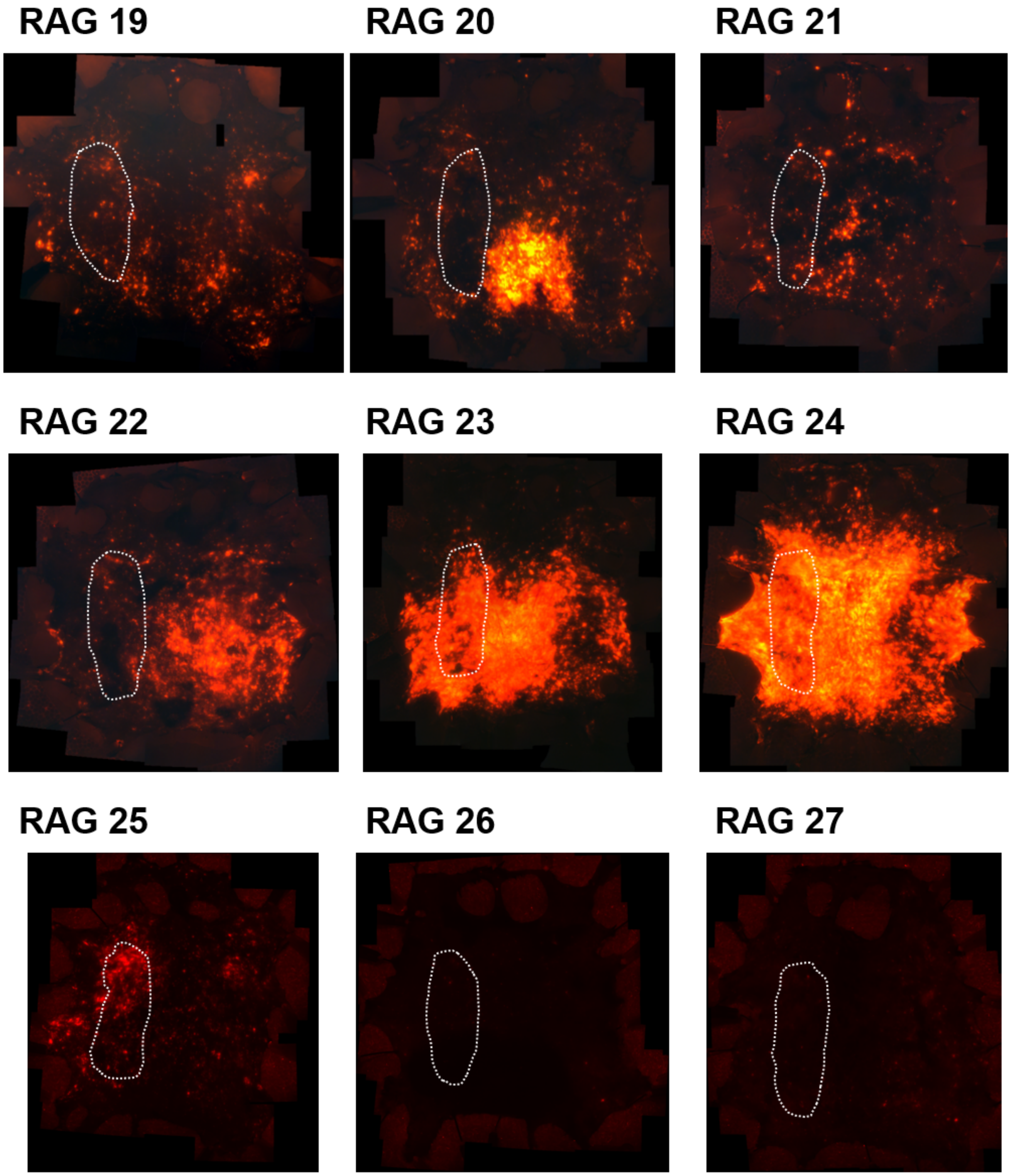

Supplementary Figure 11 | Stereomicroscopic composite images of $CD4^{+}$ T cell recipient $RAG^{-/-}$ mice

Composite images of whole mouse skins are imaged from the hypodermal side with the same settings and exposure time. Therefore, the right-side flank is on the left-hand side of images and vice versa. Red fluorescence intensity is equal to parasite density in the skin. The broken white line outlines the sand fly exposed skin area.

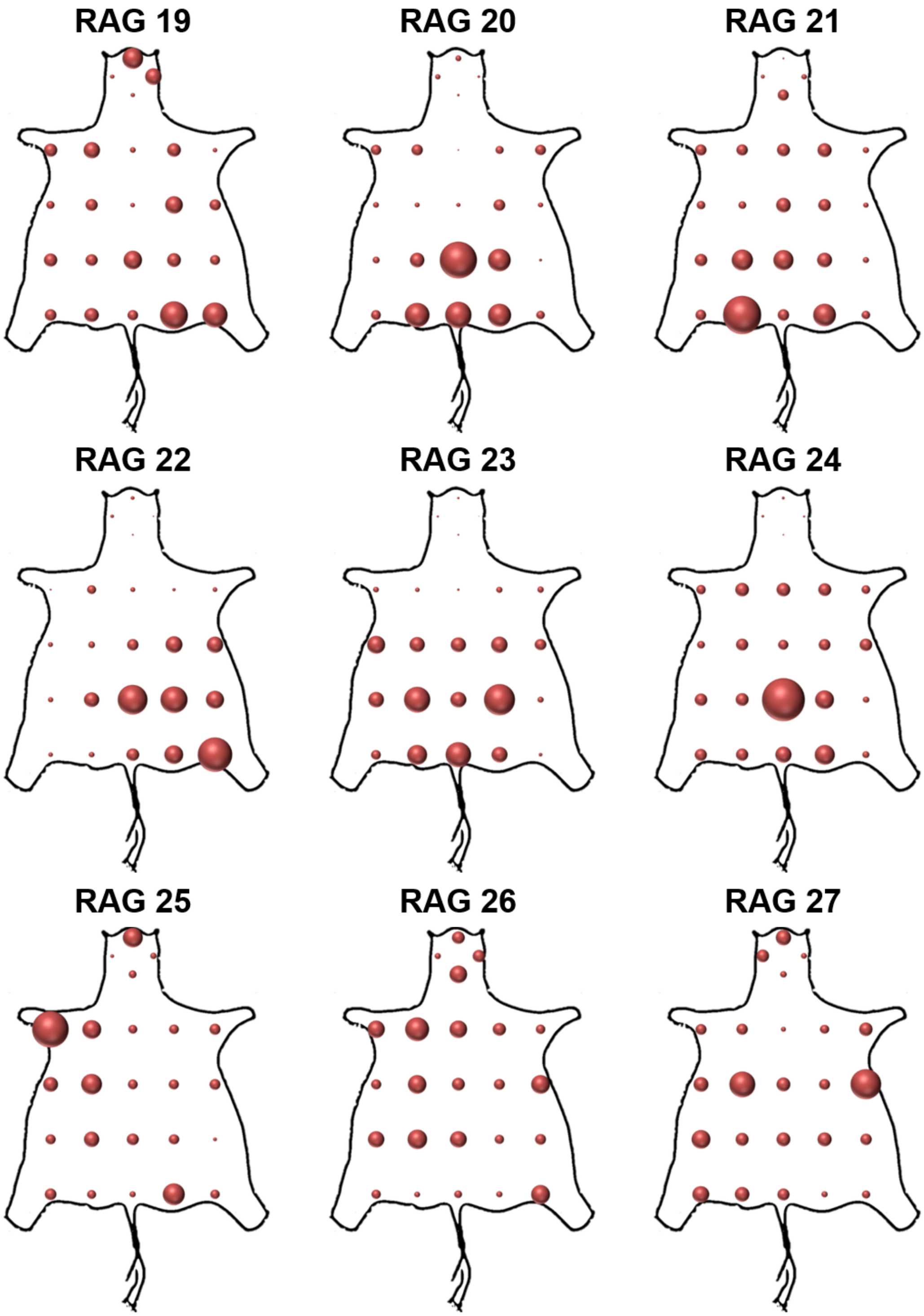

Supplementary Figure 12 | Bubble graphs of parasite load between punch biopsy sites in individual infected CD4^{+} T cell recipient RAG$^{-/-}$ mice

The size of each bubble represents the proportion of total parasite load determined by qPCR in all biopsy sites (N=24), allowing comparison between mice. Bubbles indicates distribution of parasites across the mouse skin as a means to identify preferential areas of parasite accumulation.

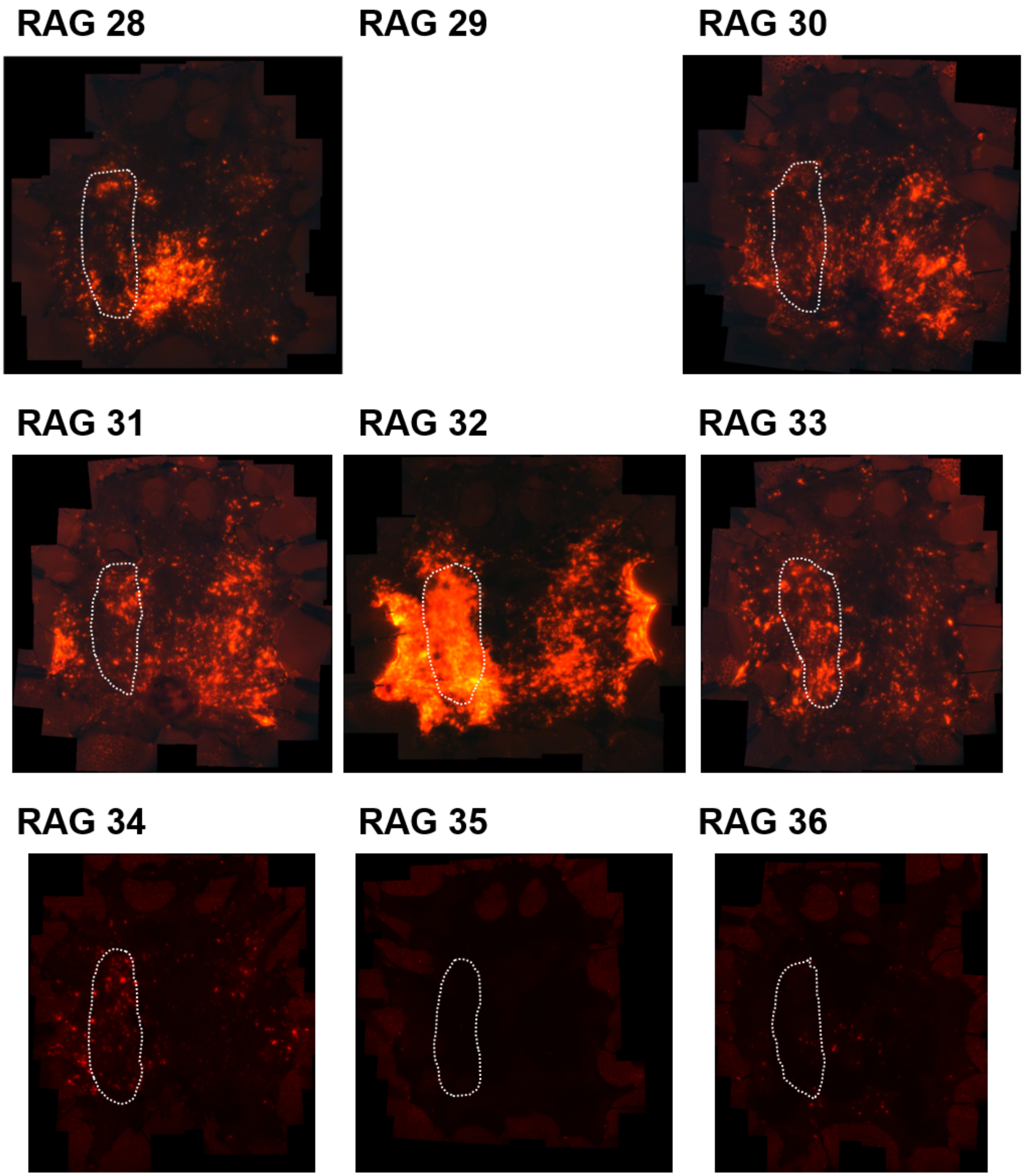

Supplementary Figure 13 | Stereomicroscopic composite images of tdTom LV9 long-term infected CD8$^+$ T cell recipient RAG$^{-/-}$ mice

Composite images of whole mouse skins are imaged from the hypodermal side with the same settings and exposure time. Therefore, the right-side flank is on the left-hand side of images and vice versa. Red fluorescence intensity is equal to parasite density in the skin. The broken white line outlines the sand fly exposed skin area. Images for Rag 29 are not available.

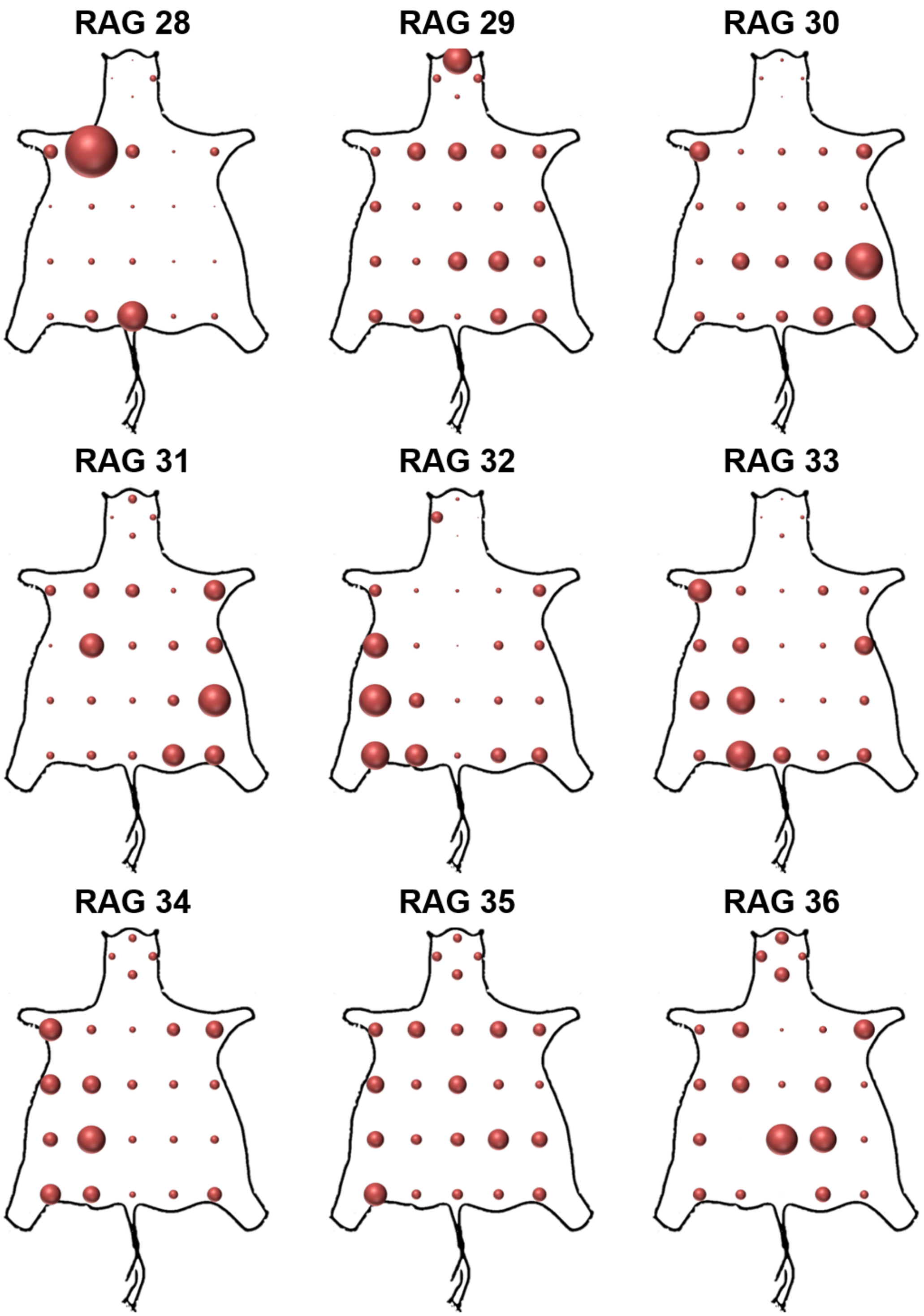

Supplementary Figure 14 | Bubble graphs of parasite load between punch biopsy sites in individual infected CD8^{+} T cell recipient RAG$^{-/-}$ mice
The size of each bubble represents the proportion of total parasite load determined by qPCR in all biopsy sites (N=24), allowing comparison between mice. Bubbles indicates distribution of parasites across the mouse skin as a means to identify preferential areas of parasite accumulation.

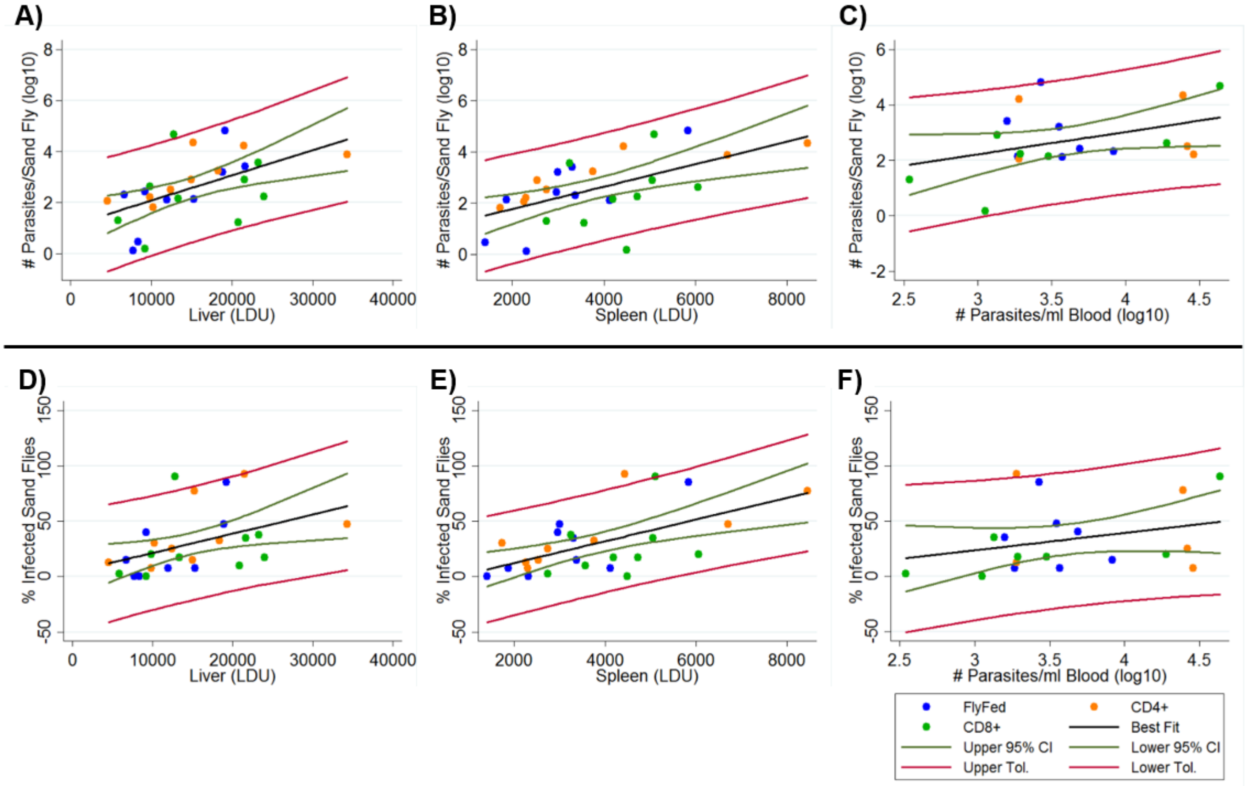

Supplementary Figure 15 | Correlating tissue parasite load to outward transmission success

This figure is an extension of Fig.3. Correlation plots comparing sand fly parasite load (determined by qPCR) with host parasite loads in (A) liver (LDU), (B) spleen (LDU) and (C) blood (qPCR). Correlation plots comparing frequency of infected sand flies with host parasite loads (D) liver (LDU), (E)spleen (LDU) and (F) blood (qPCR). Mice from all experimental groups were included in the analyses, but represented by group in the plots (see legend). The graphs show the line of best fit (black), the 95% confidence intervals (CI; green) and the tolerance bands (red). All qPCR data were $\log_{10}$-transformed. Biological replicates N=9 per group, total N=36.

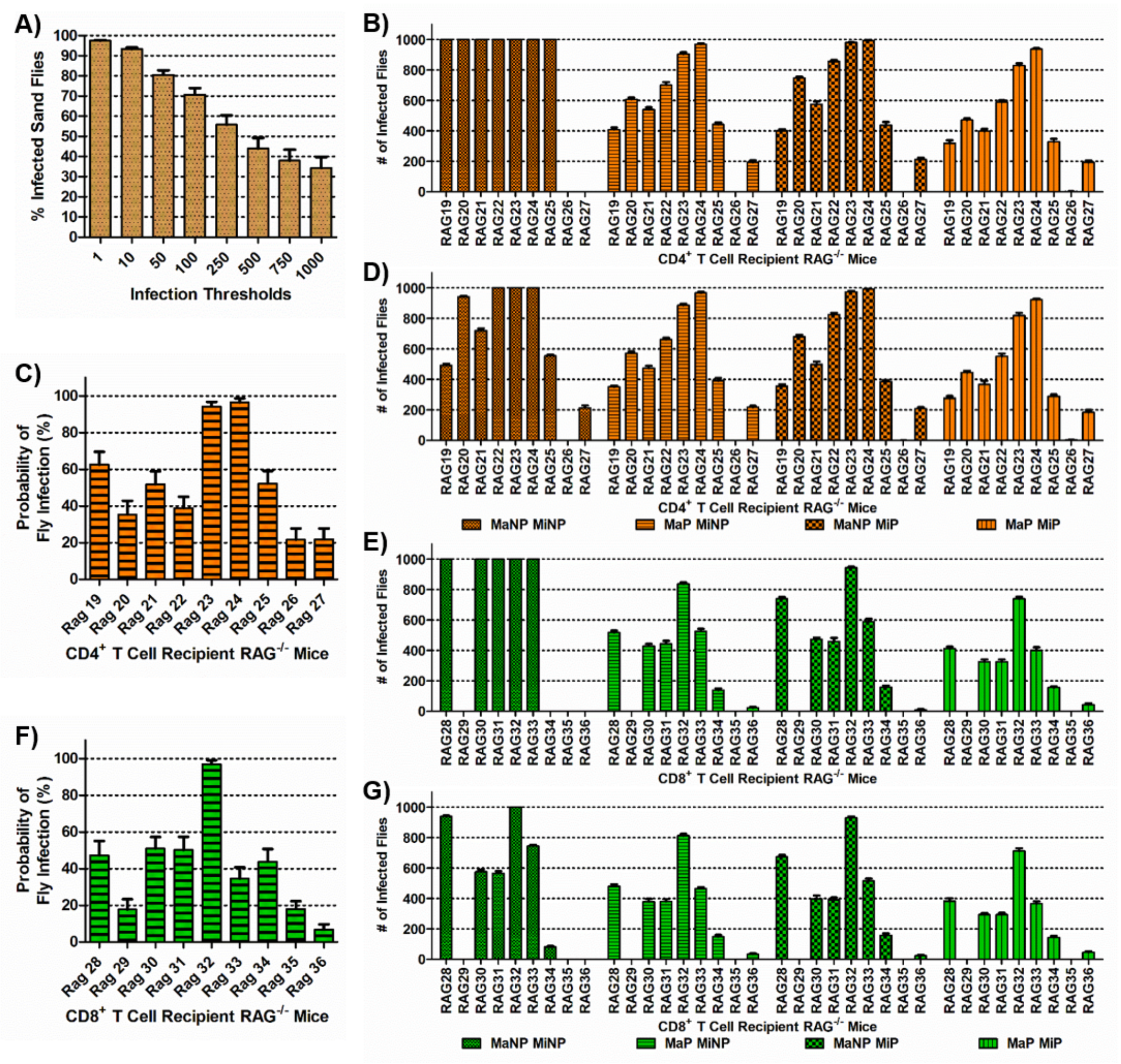

Supplementary Figure 16 | Predicting outward transmission potential

(A) Modelling the in vivo amastigote infection threshold based on the RAG$^{-/-}$ mouse skin data shown in Figure 1 by in silico reduction of amastigote infectivity. The X-axis shows assumed minimum parasite loads to establish an infection for the model. (B, E) Model predicting host infectiousness to sand flies using fixed parameters (infection threshold = 1000 amastigotes, blood pool volume = 0.19 mm^3, fly feed volume = 1.6 mm^3). Either a macro-(Ma)/micro-(Mi) scale homogeneous (MaNP MiNP; NP = non-patchy), a macro-scale patchy micro-scale homogeneous (MaP MiNP; P = patchy), a macro-scale homogeneous micro-scale patchy (MaNP MiP) or a macro-/micro-scale patchy (MaP MiP) distribution is assumed for the CD4$^+$ and CD8$^+$ T cell recipient groups, respectively. Graphs are based on 10 model iterations. Standard Error bars are shown. (C, F) Estimation of probability of sand fly infection based on experimental sand fly data for the CD4$^+$ and CD8$^+$ T cell recipient groups, respectively. Graphs are based on 10 model iterations. Standard Error bars are shown. (D, G) Model predicting host infectiousness with infection threshold reduced to 500 amastigotes, a 50% amastigote loss post ingestion and with uniformly random blood pool volumes (0.05 – 0.42 mm^3) and fly feed volumes (0.1-1.6 mm^3). Non-patchiness/patchiness as in (B, E).

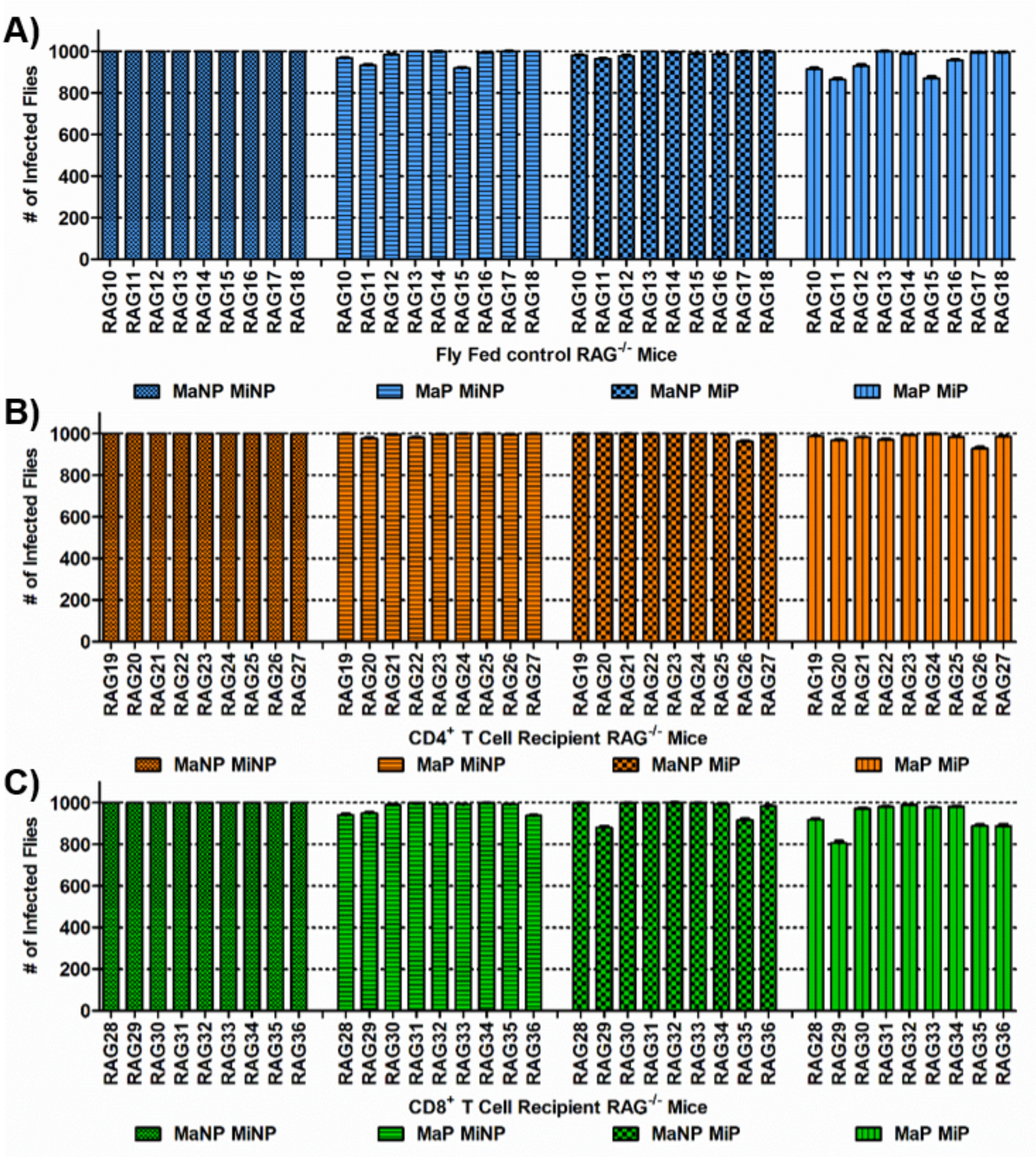

Supplementary Figure 17 | Predicted outward transmission potential using blood pool scales and no infection threshold

(A-C) The graphs represent prediction of average infection success in sand flies for the Fly Fed, $CD4^+$ and $CD8^+$ T cell recipient groups, with complete removal of the infection threshold. Graphs are based on 10 model iterations. Standard Error bars are shown.

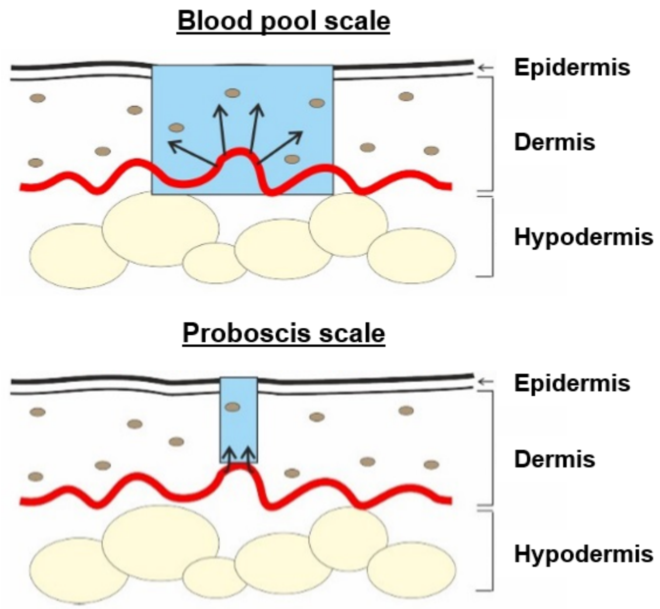

Supplementary Figure 18 | Schematic of the two respective micro-scale parasite distribution models

At the micro-scale, the model considers a contribution of skin parasites to the infectious dose ingested by sand flies at two extremes: (i) at the blood pool scale (mean: 0.19 mm^3) where all parasites falling into the blood pool contribute to the infectious dose and (ii) at the proboscis scale (mean: 0.000106 mm^3) where only parasites in the immediate vicinity of the penetrating proboscis contributed to the infectious dose from the skin. In the later model, the impact of parasitemia on the infectious doses is significantly higher, due to the considerably smaller volume of skin considered (~1792-fold small than mean blood pools).

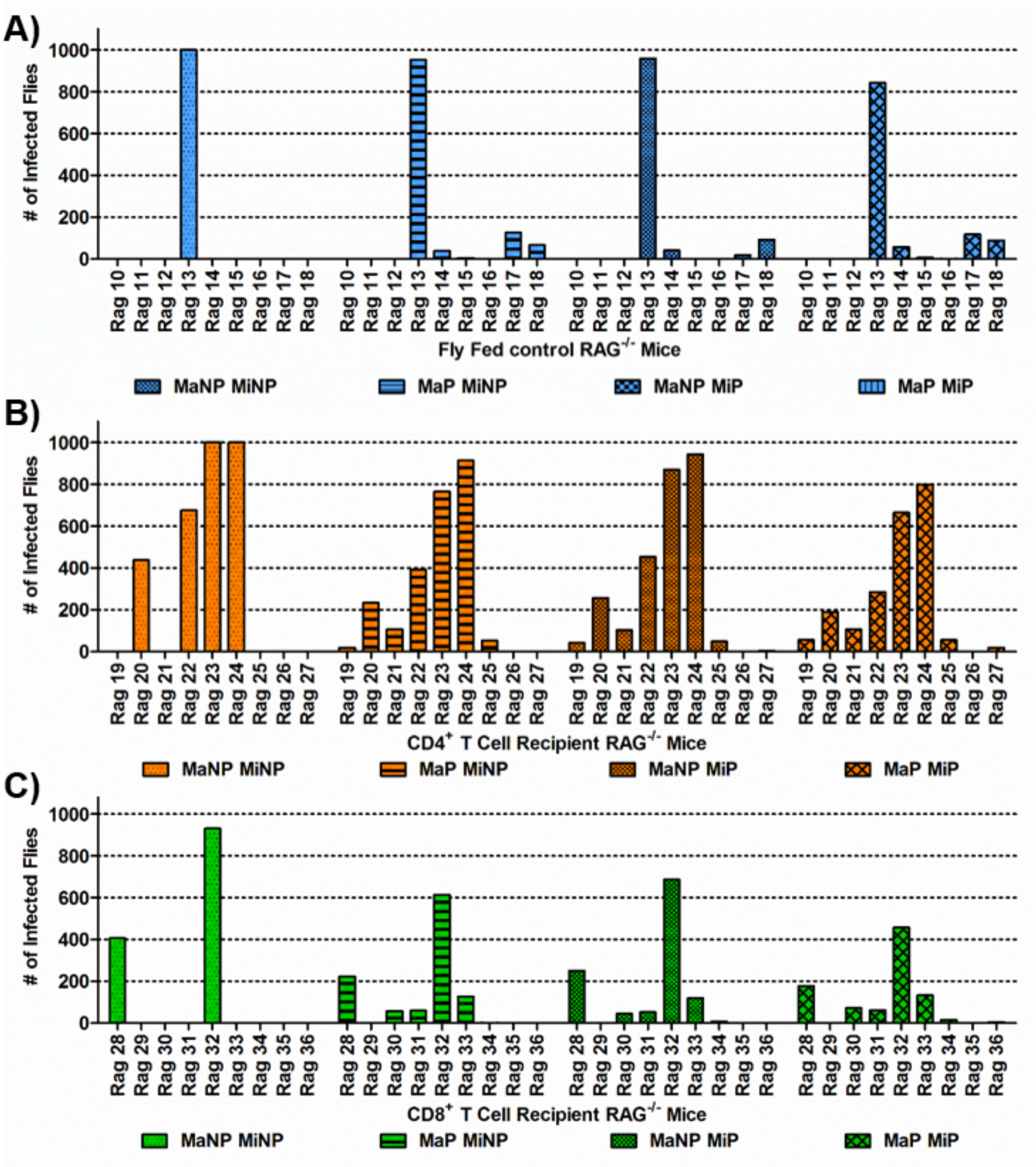

Supplementary Figure 19 | Predicting outward transmission potential using proboscis dimensions in the microscale model

(A-C) The graphs represent prediction of average infection success in sand flies for the Fly Fed, $CD4^+$ and $CD8^+$ T cell recipient groups. In this version of the model, the blood pool volume is replaced with the proboscis volume. Contributions of blood parasitemia to the infectious load are also considered. Graphs are based on 10 model iterations. Standard Error bars are shown.

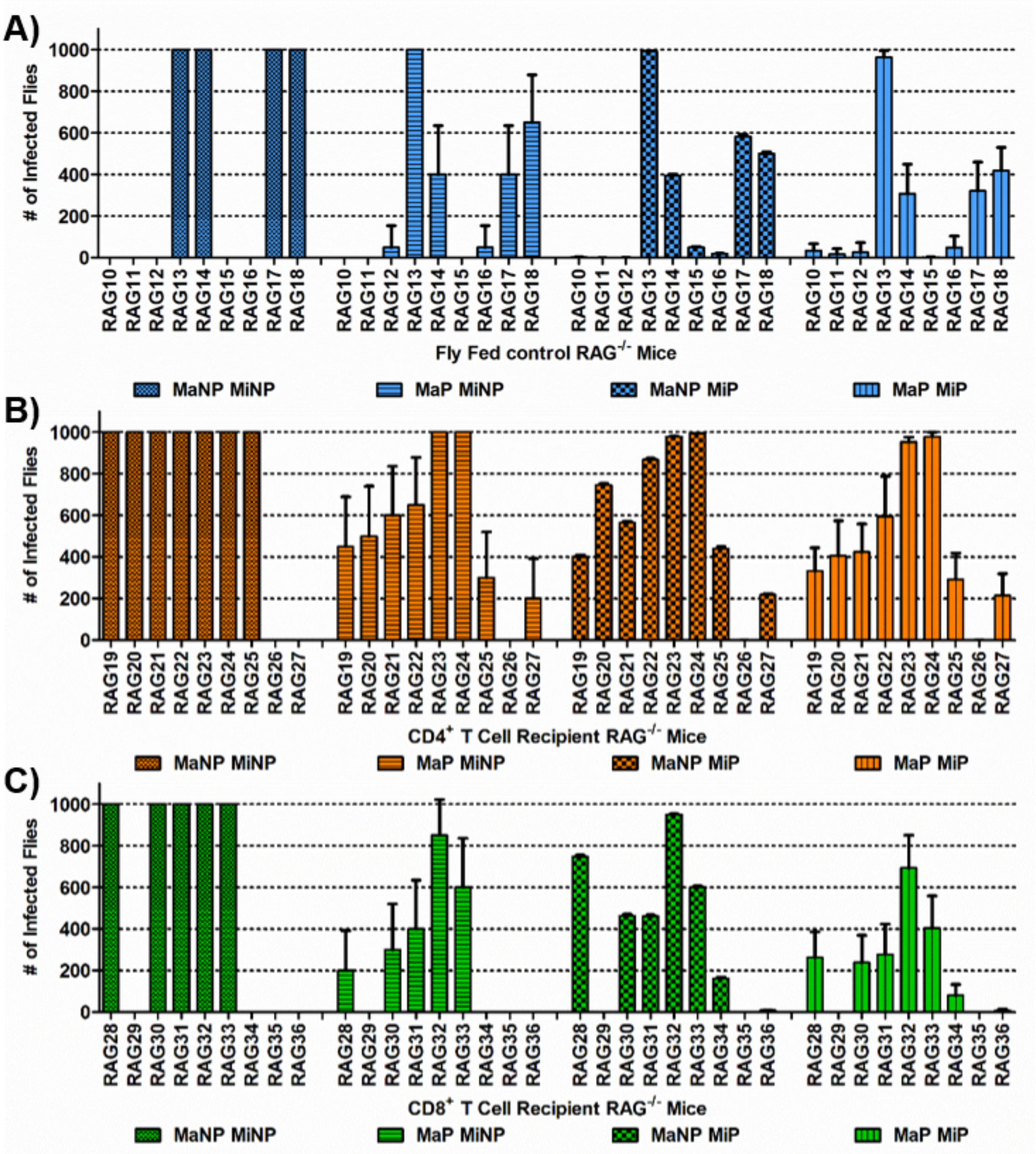

Supplementary Figure 20 | Prediction outward transmission potential using blood pool dimensions in the microscale model by bootstrapping empirical data
(A-C) The graphs represent prediction of average infection success in sand flies for the Fly Fed, $CD4^+$ and $CD8^+$ T cell recipient groups. Here, the empirical skin parasite burden data was not fitted to a distribution within the model to show that our conclusions do not rely on any extreme values within the fitted distributions. Graphs are based on 20 model iterations. Standard Error bars are shown.

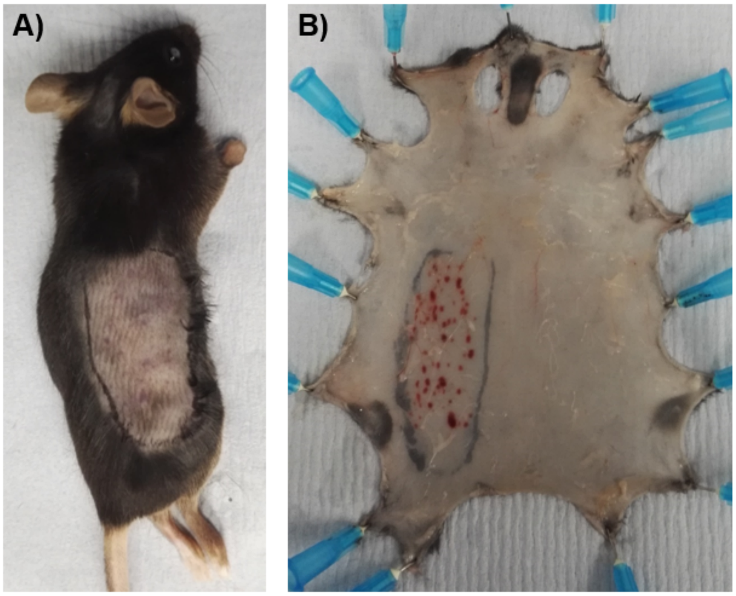

Supplementary Figure 21 | Sand fly feeding area on mouse skin

(A) An area equivalent to 10-12 punch biopsies (ø 0.8 cm) was exposed on the right flank by shaving with electrical clippers. The area was marked before sand fly exposure. Only the shaven area was exposed to sand flies. (B) Blood pools in the mouse skin inflicted by the bite of sand flies could be visualised for diameter measurement from the hypodermal face.

Supplementary Table 1 | Values for macro-scale parasite distributions in skin by Maximum Likelihood Estimations

Maximum Likelihood Estimates							
Akaike Information Criterion							
Mouse ID	Normal	Neg Bin	Exp.	Mouse ID	Normal	Neg Bin	Exp.
Rag 1	-451.7	-434.2	-436.9	Rag 19	-592.6	-576.0	-578.4
Rag 2	-459.3	-427.5	-429.6	Rag 20	-673.0	-622.7	-632.9
Rag 3	-453.8	-430.9	-433.6	Rag 21	-637.4	-598.6	-601.1
Rag 4	-361.2	-364.5	-380.2	Rag 22	-705.2	-653.5	-666.9
Rag 5	-436.8	-396.3	-398.3	Rag 23	-790.6	-752.8	-762.9
Rag 6	-568.1	-532.1	-536.0	Rag 24	-859.1	-808.9	-813.5
Rag 7	-680.7	-646.3	-651.6	Rag 25	-614.9	-580.8	-582.8
Rag 8	-645.3	-622.3	-625.1	Rag 26	-457.3	-450.5	-457.5
Rag 9	-546.7	-518.9	-522.8	Rag 27	-571.0	-549.4	-553.7
Rag 10	-513.6	-488.6	-490.8	Rag 28	-701.7	-605.7	-633.3
Rag 11	-506.5	-462.7	-465.9	Rag 29	-413.6	-396.3	-401.9
Rag 12	-526.1	-483.0	-485.2	Rag 30	-623.0	-583.3	-587.0
Rag 13	-830.2	-810.4	-812.5	Rag 31	-614.7	-584.5	-586.7
Rag 14	-601.0	-577.1	-579.7	Rag 32	-745.6	-702.7	-712.0
Rag 15	-590.3	-512.3	-524.6	Rag 33	-631.9	-601.5	-605.4
Rag 16	-534.4	-507.9	-510.8	Rag 34	-560.8	-541.3	-545.0
Rag 17	-619.0	-604.6	-607.3	Rag 35	-416.3	-407.3	-421.4
Rag 18	-594.5	-590.1	-595.2	Rag 36	-525.3	-494.7	-501.2

Model selection and parameter values for micro-scale heterogeneity. AIC scores for Normal, Negative Binomial and Exponential are provided, together with the maximum likelihood estimate for the parameter k_{Micro} characterising micro-scale parasite heterogeneity. The analysis was carried out using qPCR data from 24 punch biopsies per mouse. The value closest to zero per row indicated most appropriate parasite distribution per skin punch biopsy per mouse. The colour code corresponds to the four different mouse groups analysed in this study (grey: Untreated; blue: Fly Fed; orange: Fly Fed $CD4^+$ T cell recipient; green: Fly Fed $CD8^+$ T cell recipient) as shown in the figures. Yellow indicates the value closest to zero and with that the best fit for a distribution.

Supplementary Table 2 | Values for micro-scale parasite distributions in skin by Maximum Likelihood Estimations

Blood Pool Size			
Maximum Likelihood Estimates			
Akaike Information Criterion			
**Image ID**	**Normal**	**Neg Bin**	**Exp**
**All**	-100.5	-91.2	-95.1

Proboscis Size			
Maximum Likelihood Estimates			
Akaike Information Criterion			
**Image ID**	**Normal**	**Neg Bin**	**Exp**
**1**	-71.0	-50.6	-44.4
**2**	-	-	-
**3**	-100.8	-76.7	-80.2
**All**	-320.5	-165.8	-188.0

Model selection and parameter values for micro-scale heterogeneity. AIC scores for Normal, Negative Binomial and Exponential are provided, together with the maximum likelihood estimate for the parameter k_{Micro} characterising micro-scale parasite heterogeneity. The analysis was carried out using z-stack tile-scanning confocal microscopy from images and assumes that each blood pool is 1.5 mm in diameter. The value closest to zero per row indicated most appropriate parasite distribution per skin punch biopsy per mouse.

Supplementary Table 3 | Model parameters

Parameter	Fixed	Variable Range*	Reference
ø Punch Biopsy	0.8 cm	-	This study
Body Skin Thickness	0.24 cm	-	[40]
Punch Biopsy Vol.	12 mm^3	-	This study
ø Blood Pool	1 mm	0.5 – 1.5 mm	This study
Blood Pool Vol.	0.19 mm^3	0.05 – 0.42 mm^3	This study
ø Proboscis	29 µm	-	[41]
Proboscis length	320 µm	-	[41]
Proboscis Vol.	0.000106 mm^3	-	This study
Fly Feed Vol.	1.6 µl	0.1 – 1.6 µl	[20, 42]
Amastigote Loss	50%	-	[19]
Infection Threshold	1000	10 – 1000	[21, 22]
Infection Probability	0.75	-	[8]
Fraction of parasite population in patches (F_P)	0.8	-	This study
Fraction of skin covered by patches (F_S)	0.3	-	This study
Spatially averaged parasite density in skin (μ) in preliminary model	100 mm^{-3}	-	This study
Range of spatially averaged parasite densities in empirical data	88 – 25132 mm^{-3}	-	This study

* Variable ranges were generated by applying a uniform random function that assigned values randomly to each in silico sand fly individually.

Supplementary Note 1 | Overview of modelling framework

The mathematical models are designed to be parsimonious in that they rely on a minimal set of biologically reasonable assumptions and, where possible, use empirical data to quantify processes. The fundamental mathematics is motivated by models of predator-prey encounter rate[16]. In its simplest form, Pitchford et al. (2001) considers a single uninformed predator searching for numerous discrete prey for a fixed amount of time[16]. The predator moves at a constant speed and successfully encounters any prey which falls within a set fixed distance of the predator. The prey may be either distributed homogenously at random throughout the environment, or may be concentrated in patches. The total mean-field concentration of prey items is held constant. In the former (homogeneous) case prey are encountered as a Poisson process; the time between encounters is an exponential random variable with mean inversely proportional to the mean prey concentration. This results in a Poisson-distributed total number of prey encounters. In the latter (patchy) case, the same number of prey is encountered on average. However, the random Poisson processes governing entering and leaving patches, and of encountering prey when within patches, result in a broader distribution of the total number of prey encounters (closely related to a Negative Binomial distribution, with details depending on relative patch dimensions[16]).

These ideas are carried forward to the context of *Leishmania* transmission, with the biting sand fly as “predator” and the heterogeneously-distributed parasites as “prey”.

Supplementary Note 2 | Preliminary model

Consider a single sand fly feeding on a single mouse host, which has, on average, μ parasites mm^{-3} in its skin. The sand fly feeds at a single location, chosen at random, and ingests a fixed volume V_{FF} of blood. In the case of a homogeneous distribution of parasites throughout the skin, the sand fly will ingest a Poisson-distributed number of parasites with mean $V_{FF}\,\mu$ [16], as illustrated in Supplementary Figure 3C.

Suppose, however, that a fraction F_P of the total skin parasite population is concentrated within patches which cover a fraction F_S of the skin, with a local parasite density of μ_P. The remaining fraction ($1-F_P$) of parasites are distributed randomly throughout the remaining fraction ($1-F_S$) of skin with a local population density of μ_H. To conserve the spatially averaged parasite population density, we must have

$$(\mu_P F_S) + \mu_H (1 - F_S) = \mu . \qquad \text{(S1)}$$

Similarly, the definition of F_P requires that

$$F_P = \mu_P F_S / (\mu_P F_S + \mu_H (1 - F_S)). \qquad \text{(S2)}$$

A sand fly is assumed to feed at a random location, either within out outside of a patch. It therefore ingests a Poisson-distributed number of parasites with mean either $V_{FF}\,\mu_P$ (if it feeds in a patch i.e. with probability F_S) or with mean $V_{FF}\,\mu_H$ (if it feeds outside a patch i.e. with probability $1 - F_S$). When viewed over a large population of independent sand flies, this results in a bimodal distribution with a mean equal to μ (from (S1)), as illustrated in Supplementary Figure 3D.

The strict dichotomy of "patch" versus "non-patch" is an idealisation; the biopsy-scale samples reveal that the local density of parasites is best described using a negative

binomial distribution with the same mean μ but with variability described by a dispersion factor *k* which captures the shape (and variance, equal to μ (1 + μ / *k*)) of the empirical distribution. For very large *k* there is essentially no patchiness, resulting in a Poisson distribution as in Supplementary Figure 3C. However, the observed values of *k* range between 0.6 and 3.3 resulting in strongly heterogeneous distributions of local parasite density. The outputs in Supplementary Figure 3E are generated by assuming that each sand fly feeds at a location where the local parasite density μ_L is sampled from the empirical distribution (or from a distribution with the same mean but with a relatively low (k = 0.3) or high (k = 2.0) dispersion factor); the number of parasites ingested is then Poisson random variable with mean V_{FF} μ_L.

Supplementary Note 3 | Modelling patchiness and infection at multiple-scales

Figure 4 shows a schematic outline of the approach used for modelling patchiness at multiple scales. The principles involved match those explained in Supplementary Note 2 (above), but this time the spatially averaged parasite population density within each mouse, and the roles of heterogeneity of parasite distribution at both the macro- and micro- scales are considered.

Accordingly, for each mouse *i* a negative binomial distribution NB(μ_i, k_i) is fitted. For each sand fly feeding on mouse *i*, an independent NB(μ_i, k_i) random variable *Z* is then generated, and the number of parasites ingested by that sand fly is then simulated as a Poisson random variable with mean V_{FF} *Z*.

The results presented in Figure 5A and Supplementary Figure 16B and E extend these concepts to account for patchiness or homogeneity (P or NP) at both the macro- (Ma) and micro- (Mi) scales, as explained in the main text and repeated here for completeness. In the MaPMiP case (i.e. patchiness at both scales) for each sand fly *j* feeding on mouse *i* an independent NB(μ_i, k_i) random variable Z_i again characterises local macroscopic parasite density. Micro-scale patchiness is them modelled by a further mixture: the number of ingested parasites is a NB(Z_i, k_{Micro}) random variable (Supplementary Table 2) where k_{Micro} quantifies the micro-scale heterogeneity and the feeding at the micro-scale is assumed to be a Poisson process with a rate proportional to the local parasite density at the micro site.

Supplementary Note 4 | Threshold for infection

Within the model, it is assumed that a certain threshold of parasites must be ingested in order for the sand fly to be infected. The graphs in Figure 5A and Supplementary Figure 16B and E assume that 1000 amastigotes are needed to cause an infection. Only sand flies who ingest at least this many parasites are considered to be infectious. This is, of course, a simplification. Changing the threshold value is readily accomplished within the model (Supplementary Figure 16A). The qualitative outcomes of the model are unchanged across a wide range of plausible thresholds (Figure 5 C and Supplementary Figure 16D and G). Lowering the infection threshold to e.g. 500 amastigotes can be argued to provide a better fit to the data. In the absence of a threshold (i.e. when it is assumed that a single parasite is sufficient to cause the sand fly to be infected) the proportion of uninfected sand flies is found to be unrealistically (and uniformly) high

(Supplementary Figure 17), which does not reflect the range of observed infection probabilities as illustrated in Figure 5B and Supplementary Figure 16C and F.

Supplementary Note 5 | Simulations based on empirical data

To avoid the need to fit probability distributions, it is possible to use the identical model framework described above to carry out the simulations summarised in Figure 5 and Supplementary Figure 16, using heterogeneity based on the empirical data alone. Explicitly, a bootstrap method can be used whereby a sand fly feeding on mouse *i* is assumed to feed at a site with local parasite population density equal to that of one of the biopsy-scale locations (chosen with equal probability; Supplementary Figure 20). This captures the same variability (although necessarily at a coarser resolution) and results in qualitatively identical predictions, showing that our conclusions do not rely on any extreme values within the fitted distributions.

Supplementary References:

8. Miller, E. *et al.* Quantifying the contribution of hosts with different parasite concentrations to the transmission of visceral leishmaniasis in Ethiopia. *PLoS Negl. Trop. Dis.* **8,** e3288 (2014).

16. Pitchford, J. W. & Brindley, J. Prey patchiness, predator survival and fish recruitment. *Bull. Math. Biol.* **63,** 527–46 (2001).

19. Pimenta, P. F. P., Modi, G. B., Pereira, S. T., Shahabuddin, M. & Sacks, D. L. A novel role for the peritrophic matrix in protecting Leishmania from the hydrolytic activities of the sand fly midgut. *Parasitology* **115,** 359–369 (1997).

20. Rogers, M. E., Chance, M. L. & Bates, P. A. The role of promastigote secretory gel in the origin and transmission of the infective stage of Leishmania mexicana by the sandfly Lutzomyia longipalpis. *Parasitology* **124,** 495–507 (2002).

21. Sádlová, J. *et al.* Leishmania donovani development in Phlebotomus argentipes: comparison of promastigote- and amastigote-initiated infections. *Parasitology* 1–8 (2016). doi:10.1017/S0031182016002067

22. Anjili, C. *et al.* Estimation of the minimum number of Leishmania major amastigotes required for infecting Phlebotomus duboscqi (Diptera: Psychodidae). *East Afr. Med. J.* **83,** 68–71 (2006).

40. Sabino, C. P. *et al.* The optical properties of mouse skin in the visible and near infrared spectral regions. (2016). doi:10.1016/j.jphotobiol.2016.03.047

41. Brinson, F. J., McKeever, S. & Hagan, D. V. Comparative study of mouthparts of the phlebotomine sand flies Lutzomyia longipalpis, L. shannoni, and Phlebotomus

papatasi (Diptera: Psychodidae). *Ann. Entomol. Soc. Am.* **86,** 470–483 (1993).

42. Ready, P. D. Factors Affecting Egg Production of Laboratory-Bred Lutzomyia Longipalpis (Diptera: Psychodidae). *J. Med. Entomol.* **16,** 413–423 (1979).
